# The Lifting Balloon: Sign of a Giant Colonic Diverticulum

**DOI:** 10.5334/jbr-btr.1363

**Published:** 2017-08-04

**Authors:** Charles Edouard Heylen, Jacques Pringot, Koen Van Belle

**Affiliations:** 1 Europe Hospitals, BE

**Keywords:** Colonic, Diverticulum, Giant, Sigmoid, Balloon, Lifting

A 48-year-old male patient was referred by his general practitioner to the radiology department for evaluation of a mobile non tender abdominal mass. The patient did not report abdominal pain, nor any changes in bowel habits. His medical history included uncomplicated sigmoid diverticulitis, prostatitis and left nephrolithiasis. Previous surgeries included left kidney stone extraction. On clinical examination the abdomen was distended, soft and without tenderness, but a large tympanic mass was palpable in the left hypochondrium. Contrast-enhanced computed tomography (CT) showed a 10.4 × 8.1 cm gas-filled mass in the left hypochondrium and adjacent to the sigmoid (Figure 1A and B). The mass appear to occur at the level of a peri-diverticulitis area where no cystic mass was shown one year earlier on a CT scan (Figure 1C). The actual cystic lesion had generated the migration of the sigmoid from the left iliac fossa to the left hypochondrium (lifting balloon sign). Gastrografin enema was performed two weeks later to exclude fistulization. The exam in standing position showed focal diverticulosis adjacent to a well-defined large gas-filled cavity of 9.7 cm (Balloon sign) in the left hypochondrium (Figure 2). The lesion did not have air-fluid level or extravasation of contrast agent and it confirmed the diagnosis of a giant sigmoid diverticulum.

**Figure 1:**
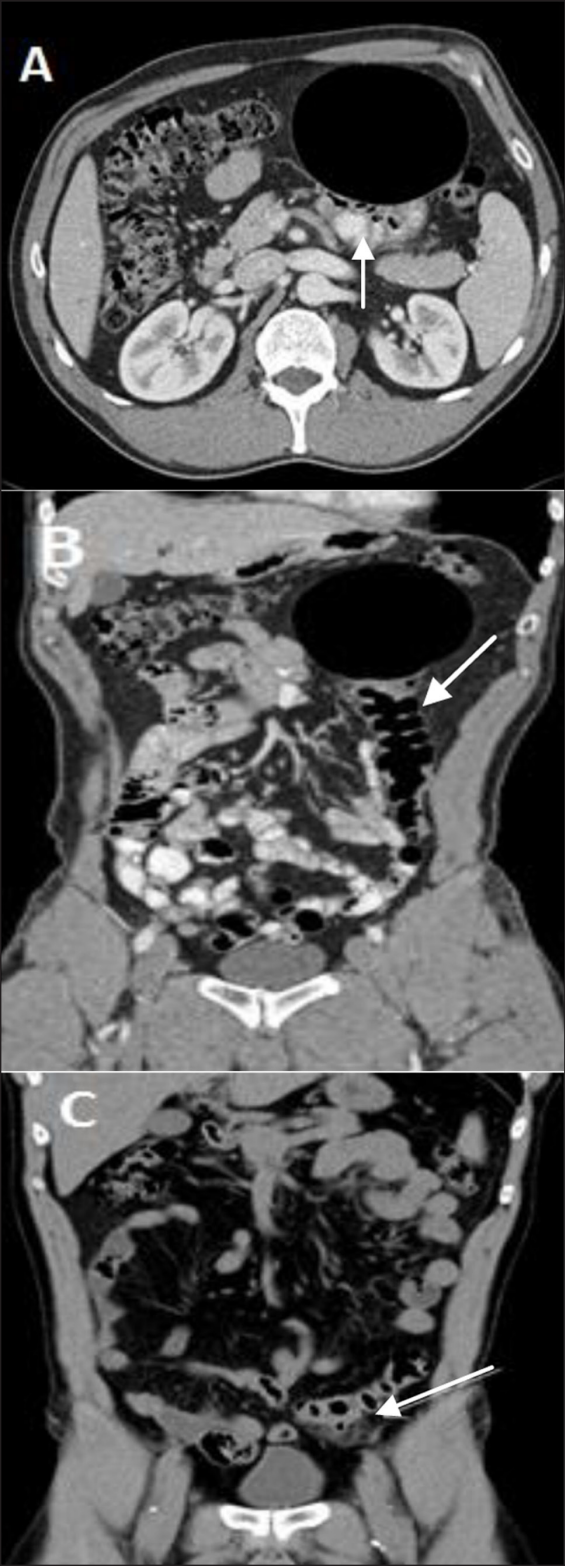
**(A)** and **(B)** Contrast-enhanced CT scan showing a 10.4 × 8.1 cm smooth-walled well-defined cystic air-filled lesion adjacent to the sigmoid (arrow) lifted in the left hypochondrium. **(C)** Unenhanced CT scan of the abdomen from one year earlier showed sigmoid diverticulitis (arrow) located in the left iliac fossa and without the cystic mass.

**Figure 2:**
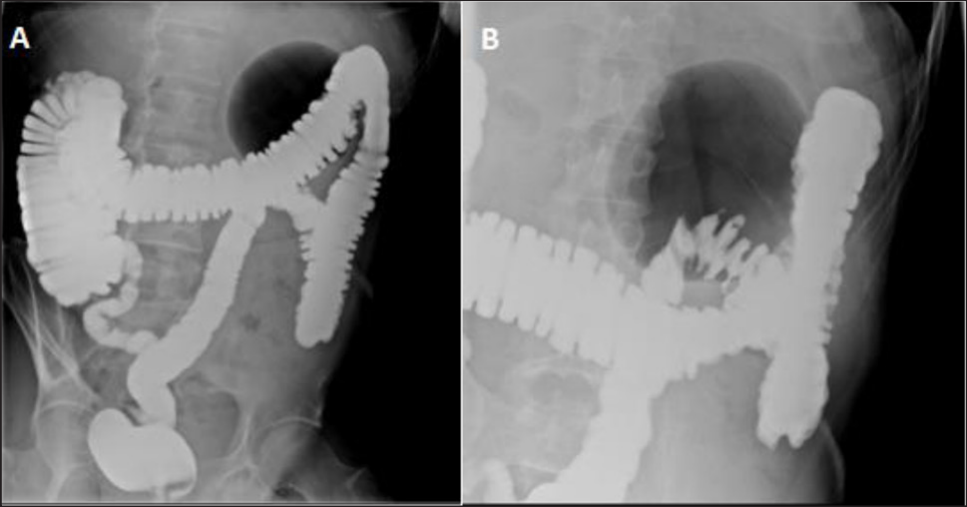
Gastrografin enema in standing position shows **(A)** a well-defined large gas-filled cavity of 9.7 cm in the left hypochondrium with neither air-fluid level nor extravasation of the contrast agent (balloon sign) and **(B)** focalized diverticular disease.

A laparoscopic resection of the diverticulum and adjacent sigmoid, with primary colonic anastomosis was performed. No postoperative complication occured and the patient was discharged on post-operative day 5. The pathology examination revealed a giant sigmoid diverticulum consisting of a fibrosclerotic wall colonized by acute and chronic inflammatory elements with a complete destruction of the endodiverticular mucosal layer and was classified type 2 according to the classification of McNutt (Figure 3). No evidence of malignancy was found.

**Figure 3:**
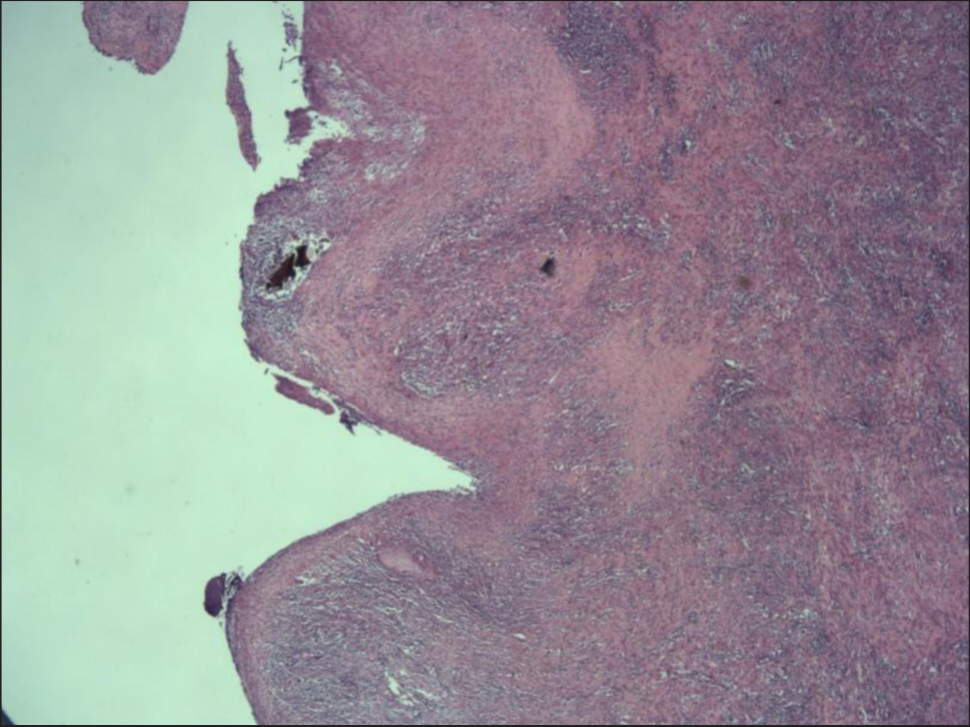
Photomicrograph (hematoxylin-eosin stain) shows the diverticulum consisting of fibrosclerotic wall colonized by mixed inflammatory elements with a complete destruction of the endodiverticular mucosal layer (McNutt type 2).

## Comment

Giant colonic/sigmoid diverticulum (GCD) is a rare complication of diverticular disease in which a diverticulum reaches 4 cm or greater in diameter. Less than 200 cases of GCD were published in the literature. GCD mostly is acquired but congenital forms have also been described. The most common clinical presentation is abdominal pain, but GCD can be asymptomatic. An abdominal mass is the most usual physical sign, but fever and abdominal tenderness can also be present. The reference and recommended examination is a computed tomography (CT) scan, which enables a correct diagnosis in nearly all cases. A smooth-walled gas-containing structure greater than 4 cm adjacent to the colon is the most common sign. GCD is not necessarily in communication with the bowel lumen. The most common complications are peritonitis caused by perforation of the GCD, abscess formation, intestinal obstruction, volvulus and infarction. A very rare complication is the development of a carcinoma.

In our case the balloon sign was associated with lifting of the sigmoid out of the lower abdomen to the left hypochondrium. Enemas/abdominal X-ray are not necessary if CT scan is available. They typically show a well-defined large gas-filled cyst, with regular and smooth walls and can present an air-fluid level if communicating with the bowel lumen. Colonoscopy is generally avoided because of the risk of perforation.

GCD are classified in three types according to the classification of McNutt et al. Type 1 diverticula are pulsion diverticula, which widen progressively, with remnants of muscularis mucosa and true muscularis, which ends at the colonic border of the diverticulum. Chronic inflammatory cells, granulation and fibrous tissue are present in its wall. Type 2 diverticula (inflammatory diverticula) are caused by a subserosal perforation, leading to an abscess cavity in the wall in communication with the bowel lumen and gradually enlarging. Their wall is composed of fibrous scar tissue, without a normal intestinal layer. Type 3 (true diverticula) consists of diverticula with all the bowel layers with a well-developed smooth muscle wall and in continuity with the bowel lumen. Type 3 diverticula usually have a congenital origin.

In our case, the giant diverticulum was a late complication of an earlier diverticulitis. According to the high incidence of complications associated with GCD, surgical treatment consisting predominantly of colonic resection with *en bloc* resection of the diverticulum is the recommended option, even if asymptomatic. Excellent results are described with null post-operative mortality and very low morbidity [1].
